# Morphology in a Parallel, Distributed, Interactive Architecture of Language Production

**DOI:** 10.3389/frai.2022.803259

**Published:** 2022-03-03

**Authors:** Vsevolod Kapatsinski

**Affiliations:** Department of Linguistics, University of Oregon, Eugene, OR, United States

**Keywords:** language production, negative feedback, parallel processing, paradigm leveling, paradigm uniformity, degrammaticalization, usage-based linguistics, interactive activation

## Abstract

How do speakers produce novel words? This programmatic paper synthesizes research in linguistics and neuroscience to argue for a parallel distributed architecture of the language system, in which distributed semantic representations activate competing form chunks in parallel. This process accounts for both the synchronic phenomenon of paradigm uniformity and the diachronic process of paradigm leveling; i.e., the shaping or reshaping of relatively infrequent forms by semantically-related forms of higher frequency. However, it also raises the question of how leveling is avoided. A negative feedback cycle is argued to be responsible. The negative feedback cycle suppresses activated form chunks with unintended semantics or connotations and allows the speaker to decide when to begin speaking. The negative feedback cycle explains away much of the evidence for paradigmatic mappings, allowing more of the grammar to be described with only direct form-meaning mappings/constructions. However, there remains an important residue of cases for which paradigmatic mappings are necessary. I show that these cases can be accounted for by spreading activation down paradigmatic associations as the source of the activation is being inhibited by negative feedback. The negative feedback cycle provides a mechanistic explanation for several phenomena in language change that have so far eluded usage-based accounts. In particular, it provides a mechanism for degrammaticalization and affix liberation (e.g., the detachment of *-holic* from the context(s) in which it occurs), explaining how chunks can gain productivity despite occurring in a single fixed context. It also provides a novel perspective on paradigm gaps. Directions for future work are outlined.

## Introduction

When asked to produce an adjective with the meaning “eligible to be disciplined,” most English speakers would produce *DIsciplinable*, with the initial stress of the base *DIscipline(d)* (Steriade, 2000), even while judging the resulting adjective a rather imperfect English word because the stress falls too far from the end (cf., *comMEND*~*comMENDable*). The goal of the present paper is to explain how novel words like *DIsciplinable* are generated, and how they are evaluated as “good enough” to attempt producing, despite their imperfections, in a brain-like, parallel, distributed, interactive activation architecture for language production.

Parallel processing with distributed semantic representations provides an account of *paradigm uniformity*, the pressure for paradigmatically related words like *discipline* and *disciplinable* to be similar in form. That is, retaining the stress of *DIscipline* in *DIsciplinable* is predicted because the two share much of their meaning, so the speaker has no choice but to activate *DIscipline* during an attempt to produce *DIsciplinable*: activation spreads to *DIscipline* from the shared semantics. This is a good thing because new words are usually produced from known words, by incorporating material from known semantically related forms. (Even *went*, the suppletive past tense of *go*, is copied from an existing, semantically-related form.) Without activating *DIscipline*, how would one generate *DIsciplinable*, and why would one assign it such an unusual, and awkward, stress pattern?

From this perspective, copying of activated long-term memory representations into a novel production plan is the main mechanism behind morphological creativity in humans (see also Kapatsinski, 2013, 2017b; Jackendoff and Audring, 2016). However, copying can also be taken too far, resulting in the diachronic process of paradigm leveling, e.g., reshaping an established plural form on the basis of the corresponding singular or vice versa (Bybee and Brewer, 1980; Tiersma, 1982).

Here, I argue that production-internal feedback, the key feature of an interactive processing architecture (Dell, 1985; McClelland and Elman, 1986), explains how speakers guard against paradigm leveling, and how they decide to change a copied base when a change is needed, or to produce a constructed form despite its imperfections. I examine the implications of a particular type of feedback mechanism, *the Negative Feedback Cycle*. In a parallel processing architecture, the intended message activates a broad range of forms that are partially compatible with the message. The Negative Feedback Cycle suppresses forms that have meanings that the speaker does not wish to express. This paper focuses on the implications of the Negative Feedback Cycle for (psycho)linguistics, but see also Chuang et al. (2020, 2021a,b) for an implemented broad-coverage model that makes use of this type of negative feedback and Jamieson et al. (2012) for a related model of associative learning.

### Assumptions

#### Processing Mechanisms

The present paper assumes that production involves parallel activation of form chunks by distributed semantic representations. The *parallel processing* perspective views the mind as a network of nodes connected by links, with activation spreading down all links connected to a node in parallel. The spread of activation is how retrieval from memory is accomplished (McClelland, 1981; Dell, 1986). Memory retrieval through parallel activation spread contrasts with serial search models of memory search often assumed in generative grammar (Yang, 2016). As is standard in psycholinguistics, I assume that the spread of activation in language production usually begins with the *message* that the speaker wishes to express (though priming can also pre-activate some form nodes). The message activates semantic/conceptual representations, which are *distributed* patterns of activation over populations of nodes in long-term memory (Hinton, 1981; McRae et al., 1997; Tyler and Moss, 2001; Rogers and McClelland, 2004). These representations are distributed in the sense that 1) each form is associated with a large number of semantic nodes, and 2) semantic overlap corresponds to node sharing. That is, forms that have similar meanings are activated by many of the same semantic nodes. Evidence for this assumption comes from many neuroimaging studies showing that individual words activate a wide range of brain areas associated with their semantics (e.g., *salt* activates the gustatory cortex; *telephone* activates the auditory cortex, *kick* activates premotor regions involved in leg movement; Hauk et al., 2008; Hoenig et al., 2008; Barros-Loscertales et al., 2012). The neuroimaging data suggest that forms can have richer and poorer semantics—some forms do not strongly activate any sensorimotor regions—and that semantic similarity is largely about the brain areas activated by a form. Thus, I will assume a semantic representation in which forms with similar semantics share activated semantic nodes, i.e., semantic representations are vectors of zeroes (nodes that a form does not activate) and positive numbers (activated nodes; here, all assumed to be equal to 1 for simplicity)^1^. The proposed architecture of planning is *interactive* in the sense that there is feedback from downstream processing units (form chunks) to upstream processing units (semantic nodes) prior to execution of the plan (Dell, 1985). Evidence for feedback is a major focus of this paper. In particular, the present paper argues for a *Negative Feedback Cycle*, which inhibits activated forms that strongly cue unintended meanings (see also Baayen et al., 2019; Chuang et al., 2021a,b).

Activation is assumed to course through the network until the speaker decides s/he has planned enough to start speaking. The activation spreading through the network during planning is assumed to be transient (in the sense of Bock and Griffin, 2000). In other words, the activation coursing through the network, as described in this paper, is assumed not to alter the connectivity structure of the network. Association weights change after the plan is constructed. This assumption is likely a simplification but it allows us to focus on processing mechanisms in this paper without addressing learning mechanisms. Once planning is complete, we assume that learning updates associations from forms to meanings, increasing the weights of associations from the semantic features of the message to the chunks forming the constructed plan, and decreasing association weights to chunks that were not selected (see Kapatsinski, 2018a; Baayen et al., 2019; Harmon and Kapatsinski, 2021; for possible mechanisms). Through learning, the system becomes and remains sensitive to the frequencies with which various semantic features co-occur with various forms in production.

#### Linguistic Theory

This paper approaches the architecture of language from the perspective of usage-based linguistic theory. Usage-based linguistics is an emergentist theory of language that considers the major goal of linguistic theory to provide mechanistic explanations for recurrent trajectories of language change, which are responsible for the emergence and change of linguistic structure (Bybee et al., 1994; Bybee, 2001, 2015). The present paper argues that a parallel distributed interactive architecture is well-suited to explaining the changes that affect morphological systems. The importance of parallel processing is well-recognized in the usage-based framework (Bybee, 1985, 2001, 2006; Bybee and McClelland, 2005). However, the role of feedback/interactivity, has not, to my knowledge, been discussed.

This focus on explaining changes is motivated by the observation that language structure is in constant flux, and the finding that the diachronic trajectories of change are far less diverse than the synchronic outcomes of change (Bybee et al., 1994; Bybee, 2003). For example, there is enormous variability in synchronic morphological systems. Some languages have very little morphology. Others have highly complex words corresponding to what would be an entire sentence in English. In some languages with complex words, the stems and affixes comprising words tend to be clearly separated like beads on a string. In others, the stems and affixes fuse together phonologically. Yet, diachronically, almost all affixes arise through a gradual process of *grammaticalization* (Bybee et al., 1994; Heine and Kuteva, 2002; Bybee, 2003). They start out as separate words and gradually fuse with surrounding words as they increase in frequency, proceeding from separate words to agglutinated discrete morphemes to synthetic markings on the stem to eventual loss (Bybee et al., 1994; Bybee, 2003). The reverse directions of change are rare or non-existent.

### The Contribution of This Paper

The present paper argues that feedback solves a number of challenges to usage-based linguistic theory. Usage-based linguistic theory has been successful in uncovering the diachronic paths of grammaticalization (Bybee et al., 1994; Heine and Kuteva, 2002), and providing mechanistic explanations for them (e.g., Bybee, 2003; Harmon and Kapatsinski, 2017). As discussed in section Parallel Processing: Form Activation = Semantic Similarity × Association Weight, these explanations crucially rely on parallel processing and distributed semantics. However, there is a well-defined class of exceptions to the directionality of grammaticalization paths, exemplified by *-ism*, which is a suffix that has developed into an independent word to mean an ideology that the speaker finds objectionable (e.g., *sexism and other isms*). The separation of *-ism* from its hosts is called *degrammaticalization* because it turns a grammatical item (a suffix) into a lexical one, a noun (Ramat, 1992). Degrammaticalization has been argued to present a major problem for usage-based views of language change, by showing that the paths of change are not unidirectional (Newmeyer, 1998; Janda, 2000). The present paper uses the Negative Feedback Cycle to provide a mechanistic usage-based account of degrammaticalization. Furthermore, by attributing degrammaticalization to feedback, which takes processing time (Dell, 1985), we can also account for the fact that degrammaticalization is far less common than grammaticalization (Ramat, 1992; Bybee, 2003).

Usage-based linguistics has argued that sublexical units emerge from generalization over experienced utterances and other units that can stand on their own, like words (Bybee, 1985, 2001). From this perspective, sublexical units like *-ism* should be able to gain autonomy when they occur in a wide variety of contexts (Bybee, 1985, 2001; Harmon and Kapatsinski, 2021). Consequently, examples in which units appear to gain autonomy despite occurring in a fixed context present a challenge to the theory. A good example is *libfixation* (Zwicky, 2010; Norde and Sippach, 2019), a process in which a string of segments becomes an affix by being “liberated” from a fixed surrounding context, as exemplified by the emergence of *-holic* from the fixed context of *alcoholic*. The present paper shows how this process too can be explained by the proposed Negative Feedback Cycle.

Architecturally, usage-based linguistics attempts to describe language using only parallel application of direct form-meaning mappings, variously called *product-oriented schemas* (Bybee, 1985), *first-order schemas* (Nesset, 2008) or *constructions* (Goldberg, 2002, 2006). I will call this the *Constructionist Hypothesis*. I call the form-meaning mappings proposed by the Constructionist Hypothesis *schematic associations*. The primary motivation for the emphasis on direct form-meaning mappings is learnability: usage-based linguistics does not posit innate linguistic knowledge (known as Universal Grammar in the competing paradigm of generative linguistics). A grammar of direct form-meaning mappings is far easier to learn from limited data than one that generates the observed forms by application of a long sequence of transformations. Therefore, adopting the Constructionist Hypothesis helps defuse arguments from the poverty of the stimulus offered in support of Universal Grammar (Bybee, 2001; Tomasello, 2003; Goldberg, 2006; Ambridge and Lieven, 2011). The present paper examines the morphological structures that appear to pose a problem for the idea that the grammar can be reduced to form-meaning mappings. In particular, I critically examine the evidence for *paradigmatic associations*, which are mappings between alternative, competing forms (Ervin, 1961). An example of a mapping thought to require a paradigmatic mapping is the [k]#_ADJ_~[s]ity#_N_ mapping in *electri*[k]~*electri*[s]*ity* and *opaque*~*opacity* (Pierrehumbert, 2006). The present paper investigates whether feedback explains away current evidence for paradigmatic associations.

In sum, this paper argues for 1) a parallel flow of activation from semantics to forms, which involves both positive and negative schematic associations (section Parallel Processing: Form Activation = Semantic Similarity × Association Weight), 2) a feedback mechanism that deactivates forms whose production is likely to lead to unintended consequences (section Feedback: Monitoring for Completion and Fixing Errors), and paradigmatic associations that carry out “repairs” of such activated forms (section Alternations as a Result of the Negative Feedback Cycle). Feedback is shown to help account for phenomena that otherwise require paradigmatic associations, limiting the range of situations in which paradigmatic associations must come into play. This is a desirable result because experimental studies show paradigmatic associations to be difficult to learn or apply (e.g., Braine et al., 1990; Smolek and Kapatsinski, 2018). On the methodological side, accepting this architecture changes what constitutes evidence for knowledge of a source-oriented, paradigmatic mapping. The proposed architecture also provides mechanistic usage-based explanations for degrammaticalization and libfixation (e.g., the liberation of *-holic* from *alcoholic*), diachronic phenomena that have not yet received a convincing mechanistic explanation from usage-based linguists (e.g., Newmeyer, 1998; Janda, 2000) and pose a challenge by.

### Related Approaches

There are several points of comparison for the proposed architecture. The primary point of comparison are morphological architectures proposed within constructionist approaches to linguistics, such as Relational Morphology (Jackendoff and Audring, 2016, 2019, 2020), Construction Morphology (Booij, 2010; Booij and Audring, 2018), Network Theory (Bybee, 1985, 2001), Word-based Morphology (Ford et al., 1997), Cognitive Grammar (Nesset, 2008), and the Entrenchment-and-Conventionalization Model (Schmid, 2020); see also Cappelle (2006), Diessel (2019, 2020) and Goldberg (2002) for related issues in syntax. These approaches take the Constructionist Hypothesis as their starting point, making use of direct form-meaning mappings. However, they vary dramatically in their position on the role of paradigmatic mappings in the grammar. Network Theory took the position that they are unnecessary, based on the empirical claim that “any morphological pattern that can be described by a source-oriented rule [i.e., a conditioned paradigmatic mapping] can also be described by a product-oriented one” (Bybee, 2001, p. 129; see also Goldberg, 2002, for syntax). However, several subsequent papers suggested that this claim does not hold, adducing evidence of productive grammatical patterns that seem to elude a product-oriented description (Pierrehumbert, 2006; Becker and Gouskova, 2016; Kapatsinski, 2017a, 2018a), endangering the Constructionist Hypothesis. Based on the existence of such patterns, all approaches to morphology mentioned above have incorporated paradigmatic mappings into the architecture of the morphological grammar. Indeed, Booij and Audring (2018) go so far as to claim that *all* productive morphology relies on paradigmatic mappings^2^.

The Negative Feedback Cycle explains away most of the evidence against the Constructionist Hypothesis, and in favor of source-oriented, paradigmatic associations (cf., Ford et al., 1997; Albright and Hayes, 2003; Pierrehumbert, 2006; Booij, 2010; Becker and Gouskova, 2016; Booij and Audring, 2018; Kapatsinski, 2018a). Therefore, the present paper forces us to reconsider what constitutes such evidence. I argue that paradigmatic associations are still needed to carry out changes to the base, and are deployed in parallel with top-down inhibition within the negative feedback cycle, when an activated form fails to match the speaker's intended message in a specific familiar way. Curiously, paradigmatic associations are likely not needed for production above the word level (see also Goldberg, 2002), which means that generating a novel word form may often be more challenging than generating an utterance from known words.

One can also compare the present proposal to computational models of morphological and lexical processing. The closest implemented model is the Linear Discriminative Lexicon (LDL) model of morphology production first proposed in Baayen et al. (2019) and subsequently extended to include interactive processing in the form of negative feedback (Heitmeier et al., 2021). The interactive version of LDL shares both direct form-meaning associations and negative feedback with the present proposal. However, a crucial difference from the present proposal is that LDL rejects paradigmatic mappings between forms.

Another point of comparison are classic interactive activation models of lexical processing (McClelland, 1981; Dell, 1985, 1986; McClelland and Elman, 1986). These models share the notion of feedback with the present proposal, and remain useful today for modeling the consequences of feedback for processing (e.g., Martin, 2007; Nozari et al., 2011; Pinet and Nozari, 2018; Nozari, 2020; Falandays et al., 2021; Magnuson et al., 2021). Because many of these models share a particular architecture, these architectural properties have become associated with the term *interactive activation*. However, many of these properties are not shared with the present proposal, as they are not inherent to interactive activation as a processing mechanism.

The term *interactive activation* here is meant only to imply that there is often feedback prior to selection as activation spreads through a network of chunks. In particular, I do not assume that the system makes use of lateral inhibition, or excitatory, positive feedback. I also do not assume that form units comprising the language network correspond to the units of formal linguistics, such as words, morphemes, or segments (see also Bybee, 2001; cf., Dell, 1986). Instead, form units emerge from linguistic experience and can be of any size. This assumption is shared with computational models of chunking (Servan-Schreiber and Anderson, 1990; Perruchet and Vintner, 1998; Solan et al., 2005; French et al., 2011; Kapatsinski, 2013; O'Donnell, 2015; McCauley and Christiansen, 2019). However, unlike interactive activation models and LDL, chunking models have not incorporated a feedback mechanism or addressed how the learned chunks interface with semantics during processing.

Like usage-based linguistics and chunking models, LDL rejects traditional linguistic units such as morphemes. However, usage-based linguists assume that form chunks are generalizations from experience, and so can be of any size. Because experience is ambiguous, many generalizations are possible, and can all coexist in a redundant, parallel system (Langacker, 1987). Furthermore, large units like words are privileged because they can occur on their own, and so little abstraction is necessary to learn them (Bybee, 1985, 2001). In contrast, LDL assumes that form chunks are small, sublexical and fixed in size (at least within a language), e.g., diphones or triphones (Baayen et al., 2019). However, this difference is not particularly crucial for the present paper, as paradigm leveling also emerges in LDL (see Baayen et al., 2019; Chuang et al., 2020, for specific examples). To illustrate, consider the production of the form *walked* using triphone form chunks. When the intended message is WALK+PAST, the correct sequence of triphones is #w*O*+*wO*k+*O*kt+kt# (where # is a word boundary). However, the meaning WALK+PAST is very similar to WALK (as most of the semantics of a verb are in the stem), so it should partially activate the trigram /*O*k#/, in addition to the context-appropriate trigrams /*O*kt/ and /kt#/. Suppose that WALK is much more frequent than WALK+PAST. The strength of activation of /*O*k#/ could then be higher than that of /*O*kt/ and /kt#/, resulting in paradigm leveling – the production of *walk* to express WALK+PAST. Feedback could help eliminate /*O*k#/ from contention because /*O*kt+kt#/ activate the intended meaning, WALK+PAST better than /*O*k#/ does. Here, I propose an activation-based mechanism for this kind of feedback, which I call the Negative Feedback Cycle.

The emphasis on direct form-meaning mappings, characteristic of usage-based linguistics and LDL, contrasts with deep learning approaches that dominate AI (see Baayen and Hendrix, 2016; Baayen et al., 2019, for discussion). One advantage of shallow architectures from a linguistic perspective is that the resulting network is interpretable, and can therefore be used for language description (e.g., Chuang et al., 2020; Caballero and Kapatsinski, 2022). Another advantage is that the architecture of the language system in the brain also appears to be relatively shallow, as estimated by the number of synaptic connections that an input has to traverse before an output is produced. Herzog et al. (2020, p. 153) write “Modern deep neural networks employ sometimes more than 100 hierarchical layers between input and output […], whereas vertebrate brains achieve high levels of performance using a much shallower hierarchy” and speculate that feedback may be one mechanism that allows for this greater efficiency of biological neural networks (cf., Beniaguev et al., 2021, for an alternative perspective). That said, many deep learning models share the architectural characteristics advocated here. In particular, both parallel processing and distributed semantic representations are widely assumed in deep learning, and feedback is actively being explored (e.g., Herzog et al., 2020).

## Planning: A Flexible Interactive Process

This section describes the proposed architecture of the production system. I begin by examining the consequences of parallel processing with distributed semantics for lexical selection and show how it results in paradigm leveling (Parallel Processing: Form Activation = Semantic Similarity × Association Weight). I then introduce the negative feedback cycle as a core part of the production system that allows the speaker to decide to initiate execution (when the plan is good enough) or delay it (when it is not), and suppresses activated forms whose production would likely have unintended consequences, while allowing them to activate more appropriate forms through paradigmatic associations (Feedback: Monitoring for Completion and Fixing Errors). The evidence for paradigmatic associations is then reconsidered in light of the fact that the negative feedback cycle explains away many findings that have been argued to support such associations (The Role of Paradigmatic Associations in Production). These sections can be seen as describing a sequence of overlapping stages in producing a form.

### Parallel Processing: Form Activation = Semantic Similarity **×** Association Weight

Given distributed semantic representations and parallel spread of activation, a multitude of forms partially matching the intended meaning must become activated. Evidence for this process comes from several findings. First, production of a word is harder if the word has many semantic competitors (e.g., Schnadt, 2009; Harmon and Kapatsinski, 2015; Rabovsky et al., 2016). Second, production of a word is harder if you have recently produced its semantic competitor (Maess et al., 2002; Marsolek et al., 2006). Third, production is harder when a semantic competitor is primed through perceptual presentation (e.g., superimposed over the picture of the target concept to be named; Meyer, 1996; Abdel Rahman and Aristei, 2010), with interference increasing with increasing semantic similarity between the competitor and the target (Rose et al., 2019). Sometimes, priming the form of a semantic competitor can even result in the erroneous production of that competitor (e.g., producing *nun* to name the picture of a priest after reading *none*; Ferreira and Griffin, 2003). Fourth, when match to semantics is controlled experimentally, speakers produce frequent words rather than their infrequent competitors (Harmon and Kapatsinski, 2017; Koranda et al., 2018). Leveling accessibility differences between the forms of frequent and infrequent words eliminates the preference to produce the frequent word, indicating that this preference is due to the influence of frequency on form accessibility (Harmon and Kapatsinski, 2017).

In a parallel processing system with distributed semantic representations, the activation that a form receives from an intended message must be influenced by at least two factors: 1) how much the intended message activates the semantics associated with the form, i.e., *Semantic Overlap* between the message and the meaning of the form (*a*_*s*_), and 2) the strength of the association from the activated semantic features to the form (*Semantic Cue Weight*). The activation of a semantic node, *a*_*s*_, determines how much activation is available to spread out of it to the associated forms. The amount actually received by any one form is then the product of *a*_s_ and the strength of the connection from *s* to the form in question (*w*_*s*→*f*_). As shown in (1), the total activation received by a form is then the sum of these products across all activated semantic nodes.


(1) $$\begin{array}{r} a_{f}=\underset{s}{\sum }a_{s}w_{s\to f}. \end{array}$$

The association weight *w*_*s*→*f*_ must, at a minimum, increase with the probability of the form (*f* ) given the semantic feature (*s*), i.e., *p*(*f* |*s*). For a form that always has a certain semantic feature, *w*_*s*→*f*_ therefore increases with the form's token frequency. Note that (1) is compatible with any architecture of production that makes use of distributed semantic representations and parallel processing, regardless of one's position on any of the controversial questions in language production, such as the nature of the form representations, the existence of online competition or cooperation between alternative forms, cascading activation and feedback. It describes the amount of feedforward flow of activation from semantics to the form level, which is assumed by every parallel model of production.

Despite the uncontroversial nature of (1) within a parallel processing framework, the formula has several interesting consequences. First, the greater the number of activated features that belong to the semantic representation associated with a form, the higher *a*_S_ and so the more strongly that form will be activated. Other things being equal, this favors semantically specific/rich forms, which indeed appear to be easier to produce in picture naming tasks (Rose et al., 2019). The consequence for morphological production is that larger, semantically richer chunks will be favored over smaller chunks, resulting in blocking/pre-emption (Aronoff, 1976). For example, the chunk *went* would receive activation from both GO and PAST, whereas *go* and *-ed* would receive activation from only one feature each. This gives memorized irregulars a leg up over regularizations because regularizations are combinations of forms. It also gives a stored executable form an advantage over computation. Second, given two forms that are equally compatible with a meaning, the more frequent form will tend to be chosen, allowing the frequency difference to be maintained. Third, blocking/pre-emption is not always effective: frequent forms can outcompete infrequent forms for production even when the infrequent form would be a better cue to the intended meaning, as shown experimentally by Harmon and Kapatsinski (2017) and Koranda et al. (2018). This can happen when there is substantial semantic overlap between the frequent form and the intended message, so that the frequent form receives activation from the intended semantics. The choice of frequent forms over semantically-similar competitors can lead frequent forms to expand in their range of uses. As argued in Harmon and Kapatsinski (2017), this is the primary driving force behind grammaticalization, resulting in forms with highly complex sets of related functions, such as those of the frequent English verb *get*.

Parallel spread of activation from distributed semantic representations, as described by (1), also explains the existence of paradigm leveling, observed in both language change (Bybee and Brewer, 1980) and language acquisition (Hoeffner and McClelland, 1993). A well-known example of leveling in language change is the leveling of singular/plural stem alternations in Frisian. In most cases, the stem of the singular has been extended to the plural. For example, *miell*~*mjillen* has become *miell*~*mielen* 'meal~meals' (Tiersma, 1982). Importantly, Tiersma has shown that in just those nouns that refer to objects that come in pairs or multiples, and where the plural is therefore likely to be more frequent than the singular, the plural stem was extended to the singular. Thus, *hoas*~*vjazzen*, “stocking~stockings” has become *vjazze*~*vjazzen*. Numerous examples from other languages can also be provided (see Tiersma, 1982). Furthermore, within a particular paradigm cell, such as the plural, rare words succumb to leveling before frequent forms (Bybee and Brewer, 1980). This favoring of the more frequent form is exactly what is expected under (1), assuming that the association strength between a semantic feature and a form increases with the frequency of that form-meaning pairing. The frequencies of semantic features, which influence their resting activation levels, likely matter as well: forms in frequent paradigm cells are less likely to succumb to leveling, even when the forms themselves are not frequent (Bybee and Brewer, 1980; Tiersma, 1982; Albright, 2008). Bybee and Brewer (1980) have further pointed out that semantic overlap also matters in the expected way: the more similar two forms are in meaning, the more likely they are to influence each other in paradigm leveling. Hoeffner and McClelland (1993) have shown that parallel activation of morphologically-related forms from shared semantics can also explain the paradigm leveling that occurs in child language acquisition, where children (particularly, those with developmental language delay, previously called specific language impairment) substitute frequent base forms for less frequent inflected forms (see also Freudenthal et al., 2021; Harmon et al., 2021).

**Figure 1:**
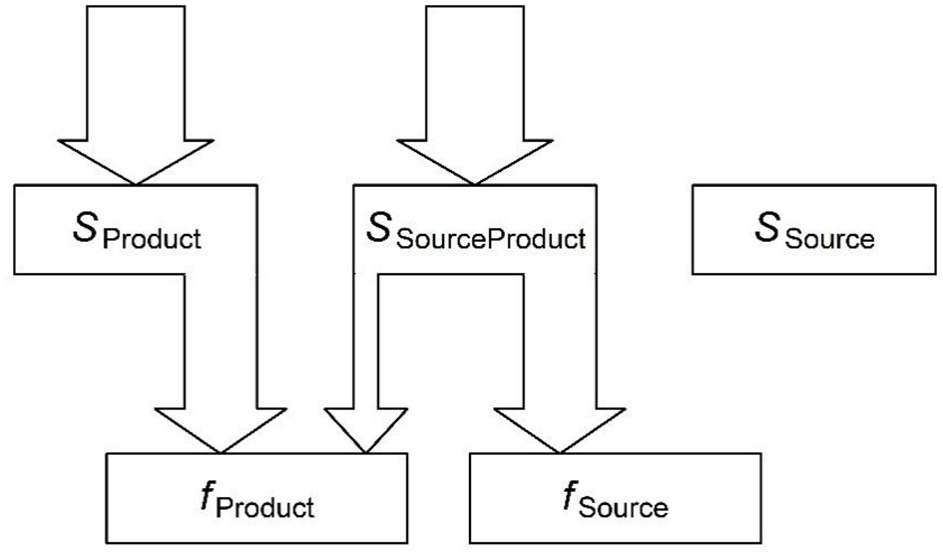
The initial, feedforward flow of activation. *s* = semantics. *f* = form. Input activation from the message shown by sourceless arrows at the top, which activate the semantic vector corresponding to the product form that is the best match to the intended message. The activated semantics are partly shared with the source form (*s*_SourceProduct_) and partly not (*s*_Product_). The shared semantics activate both forms in proportion to how well this vector predicts each form, with more activation reaching the frequent form compared to the less frequent product form (arrow width). This imbalance is what creates paradigm leveling. The additional activation received by *f*_Product_ from the meaning not shared with *f*_Source_ prevents paradigm leveling. Paradigm leveling occurs if *f*_Source_ is activated more than *f*_Product_^3^.

Figure 1 illustrates how paradigm leveling arises from parallel spread of activation from distributed semantic representations. The form that fully matches the intended semantics is indicated by *f*_Product_. This is the form that the speaker intends to produce. However, some of the semantics shared by the product form and the intended message are also shared with a *source* form, which provides source material for leveling, or for reconstructing an inaccessible form. The shared semantics are indicated by *s*_SourceProduct_. Because semantic representations are distributed, *s*_SourceProduct_ activates both forms. Because the source form, *f*_Source_, is more frequent than the product form, *f*_Product_, it has a stronger association with the shared semantics (Kapatsinski and Harmon, 2017), and so receives more activation from it (as indicated by arrow width). The product form is favored by the part of the meaning not shared with the source form (*s*_Product_), but occasionally this may not be enough, and the source form may be produced instead, leveling the infrequent form (as in Tiersma, 1982). The activation received from *s*_Product_ is greater if *s*_Product_ consists of frequent features with a high resting activation level (*a*_s_), i.e., if *f*_Product_ belongs to a frequent paradigm cell, it is more resistant to leveling. The number of *s*_Product_ features also favors *f*_Product_ over *f*_Source_, just as the number of shared features favors the more frequent *f*_Source_. Thus, leveling is most likely when *f*_Source_ and *f*_Product_ are semantically similar (as shown by Bybee and Brewer, 1980).

Because the source and product *word*forms contain smaller sublexical chunks, it is not necessarily the case that the full source wordform would replace the product wordform. For example, the Frisian plural suffix (*-en*) would likely not be associated with the shared semantics of “stocking” and “stockings” as strongly as the plural stem allomorph of “stocking” is (*vjaz*), because the suffix frequently occurs without the “stocking” meaning. As a result, the plural stem allomorph is more likely to be produced than the plural suffix when the singular form of “stocking” is intended, resulting in the pattern of leveling observed in Frisian (*hoas*~*vjazzen* > *vjaz*~*vjazzen*, and not *vjazzen*~*vjazzen*). At the same time, the proposed framework does not prohibit copying aspects of the base that are not part of the shared stem; it just makes this less likely than copying the stem. It is therefore consistent with the evidence that paradigmatic morphology can preserve non-stem aspects of the base, which eludes compositional approaches to morphology (see Booij, 1997; Ford et al., 1997).

Importantly, two forms can be coactivated without being in a particular morphological relationship. The architecture above predicts that such forms can also interfere with each other's production, resulting in leveling of some differences between them. An example of this type of leveling is the numeral four anticipating the /f/ of five (citing Osthoff and Brugman, 1878; Downing et al., 2005).

Furthermore, production in this architecture is opportunistic: whatever forms partially match the intended meaning are activated and can influence the product. These forms need not always come from the same paradigm cells. Because forms that are semantically similar to the product are more likely to interfere with its production, a close semantic competitor can preempt competition from a more distant one. Therefore, the sources of leveling usually come from semantically closest paradigm cells to the product. However, as expected from a parallel system, farther-away cells nonetheless have a detectable effect (Breiss, 2021). Furthermore, when a close cell is empty, a normally less effective competitor can become more influential because it will be activated more than any other potential source form. Hale et al. (1998) argue that this happens in the derivation of truncated hypocoristics in New York City English. These hypocoristics have the form CVC, as in *Sarah*→*S*[æ]*r*. Benua (1997) pointed out that the vowel normally comes from the corresponding CVCy hypocoristic, which is semantically closer to the truncated hypocoristics than the full form is. Thus, *Laurence*/*Larry* is truncated to *L*[æ]*r* and not *L*[*O*]*r*, matching the *-y* hypochoristic. Benua therefore proposed that speakers have a paradigm uniformity constraint demanding copying of the vowel from *-y* form into the truncated form. Hale et al. (1998) argue against this analysis because *Sarah* becomes *S*[æ]*r* despite the absence of *Sarry*. The proposed architecture is able to capture both the faithfulness of the truncated form to *Larry* rather than *Laurence* and the faithfulness of *Sar* to *Sarah*. In both cases, the truncated form retains the vowel of the semantically closest form.

### Feedback: Monitoring for Completion and Fixing Errors

The goal of planning is to settle on a sequence of actions that would express the intended message (or something close to it), and be easy to execute. Because the speaker usually faces some time pressure, and memory is limited, it is often not possible to plan the entire utterance in advance, or at least not in full articulatory detail (Meyer, 1996). As a result, the onset of execution is the result of a context-sensitive decision-making process to start speaking. For example, when the speaker faces competition for floor, they start speaking more quickly (Holler et al., 2021).

How does the speaker decide to begin execution? Motley et al. (1982) showed that production of word sequences like *hit shed*, which would result in a taboo utterance from the common phonological error of onset exchange, elicits sweaty palms (measured by the galvanic skin response) and longer planning latencies, even when the speakers are not consciously aware of the possibility for error. These results indicated an internal monitoring process that can detect that a grievous mistake is about to occur. I suggest that this process continuously adjusts the likelihood of a decision to begin execution. This proposal aligns with the idea that conflict monitoring is used to decide to engage top-down control (Botvinick et al., 2001; Nozari et al., 2011), except that in constructing a form top-down control is already engaged and the speaker needs to decide when to *dis*engage it. The Negative Feedback Cycle is intended to help make this decision.

#### The Negative Feedback Cycle

**Figure 2:**
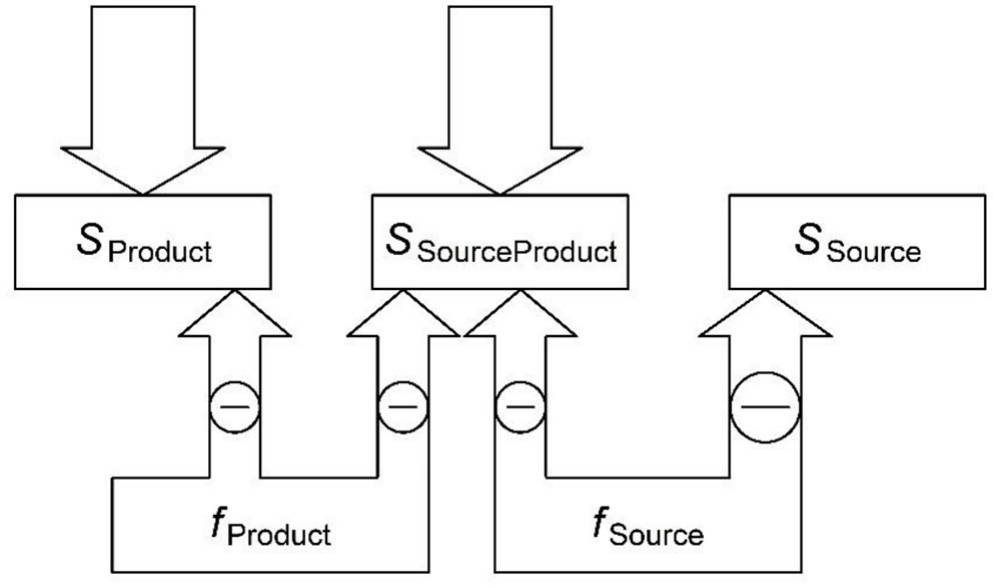
The second stage, negative feedback: the activated forms inhibit associated semantics (inhibition is shown by the circled minus signs). The amount of inhibition reaching a semantic feature from a form (and then available to spread back to the form) is proportional to the strength of the connection from the form to the semantic feature. In this example, *f*_Source_ is a strong cue to unintended semantics, *S*_Source_, which is not part of the message. After feedback, the amount of activation spreading down from an *S* vector to a form is the difference between the top-down excitation *S* is receiving from the message (sourceless arrows) and the amount of inhibition it is receiving from the forms. Here, *S*_Product_ is receiving much more excitation than inhibition and will continue exciting *f*_Product._ *S*_SourceProduct_ is receiving approximately equal excitation and inhibition and will no longer be a strong factor in form activation. The inhibition received by *S*_SourceProduct_ helps *f*_Product_ over *f*_Source_ because *f*_Source_ is favored by *S*_SourceProduct_ due to its higher frequency. Finally, *S*_Source_ is only receiving inhibition, and will begin inhibiting *f*_Source_^5^.

To begin execution of a plan, the speaker must think that the plan fits the intended message well-enough (given the time available, and the consequences of error). Therefore, the production system needs a way to determine how good the fit is. I propose that it calculates the difference between the semantic pattern of activation corresponding to the intended message and the pattern of activation elicited by feedback from the planned form (see also Baayen et al., 2019). This is accomplished by the *Negative Feedback Cycle*, which inverts the feedback coming from the planned form to the semantic level and then adds it to the current activation of the semantic nodes, which came from the message (Figure 2; see Jamieson et al., 2012, for a similar inverted feedback mechanism in associative learning)^4^.

To be more specific, negative feedback must detect differences between the intended message and the message the form is likely to transmit. It must then suppress forms to the extent that their production would have unintended consequences. According to (1), a chunk's initial activation is $a_{f_{1}}=\underset{s_{+}}{\sum }a_{s_{+}}w_{s_{+}\to f}$, where the ‘+' marks message features as intended. Some of this activation is then fed back as inhibition of the unintended message features. The amount of activation a chunk transmits to the semantics is $\underset{s}{\sum }\left(-w_{f\to s}a_{f_{1}}\right)$, where the minus turns the activation inhibitory and *w*_*f*→*s*_ is how strongly the chunk cues a feature. The weight *w*_*f*→*s*_ comes at least in part from learning during comprehension (i.e., prediction of meanings given forms).

The activation of a semantic feature after the negative feedback is $a_{s_{2}}=a_{s_{1}}-\underset{f}{\sum }\left(w_{f\to s}a_{f_{1}}\right)$, where *a*_*s*_1__is its activation level of the feature prior to the negative feedback. Some of the semantic features associated with a form are intended and others are not. Because the activation coming from the form is inverted, the feedback inhibits all semantic features associated with a form. However, intended features shared with the source form (*s*_SourceProduct_ in Figure 2) are receiving excitation from the message, preventing the inhibition from turning their overall activation negative^6^. Unintended features, in contrast, do not receive any excitation, and so end up with a negative activation level. This negative activation then passes down to the associated forms, reducing their activation levels: $a_{f_{2}}=a_{f_{1}}+\sum_{s}\left(a_{s_{2}}w_{f\to s}\right)$^7^.

Negative feedback produces a signal to begin or delay execution. Specifically, a monitor node could be connected by inhibitory links to the semantic (*s*) level (Botvinick et al., 2001). After feedback, its activation olevel would then be proportional to the overall level of inhibition across the *s* nodes ($-\underset{s}{\sum }a_{s_{2}}$). If this level is high, unintended consequences of production are likely, and the speaker should continue planning, delaying execution. As less time is left to plan, the threshold for mismatch activity level necessary to delay execution can increase, reducing the likelihood of delay. As mistakes become more costly, it can be reduced, increasing delay likelihood (see Botvinick et al., 2001; Nozari et al., 2011; Nozari, 2020; for related ideas).

#### Alternations as a Result of the Negative Feedback Cycle

Negative feedback results in a vector of differences between the intended message and the unintended message. That is, unintended features have negative activation levels, and intended ones have positive activation levels. I propose that the pattern of activations can become associated with specific repairs. Associating repairs with differences between the intended message and the planned form can account for the production of arbitrary alternations without need for paradigmatic associations (cf., Pierrehumbert, 2006; Becker and Gouskova, 2016).

For example, suppose that you are provided with the novel adjective (A) *compenturic* and asked to produce a noun (N) from it in a spoken wug test. You activate *-ity* from the meaning N (N → …*ity*). The chunk #*compenturic*# is also active and blended with …*ity*#, aligning the word boundaries, producing *compenturi*[k]*ity* (Kapatsinski, 2013). How does the speaker then avoid executing this plan? If they monitor for semantic mismatch, they can detect that feedback from *i*[k]… activates A at the semantic level, which mismatches the intended N. Note that by inverting the activations coming from the form, and then combining them with activations that had already come from the meaning, the speaker automatically comes to know what chunks need to be activated or inhibited. Activation of the intended N is strongly positive and activation of the unintended A is negative. Therefore, A would pass inhibition to form chunks associated with it, while N would pass excitation to the associated chunks. That is, the repair can be accomplished simply by interactive activation flow, coupled with inhibitory feedback to the semantic layer.

Notationally, we can summarize this process as {N; ^*^A} → {–ik/__#; +i[s]ity/__#}, where curly brackets enclose sets of nodes in the same layer (form or meaning). In other words, when the speaker intends to produce a noun, and is about to produce an adjective, this problem is solved by inhibiting chunks associated with A, such as (*i*[k]) and activating the chunks associated with N, such as *icity*#.

Let us briefly consider another example. Becker and Gouskova (2016) have shown that Russian nouns are more likely to undergo a process of vowel deletion in the Genitive singular if the nominative form has the shape (CV)CCVC rather than (CV)CVCC, e.g., *kostjor*~*kostra* but *osjotr*~*osjetra* (the *jo*~*je* alternation is a consequence of stress shifting to the suffix in the Genitive). Becker and Gouskova (2016) argued that, because deletion would result in the same Genitive shape (CV)CCCa, the preference to delete the vowel in a CVCC base compared to a CCVC base could not be accounted for by schematic associations of the Genitive, requiring the generalization that /^j^o/ is more likely to be deleted in CCVC bases. However, in the present framework, this requires simply a negative schematic mapping. Suppose that the speaker is provided with a nominative form like *kostjor*. That form activates Nominative, but the speaker is asked to produce Genitive. They can learn that in such a situation, {^*^Nom; Gen}, a /^j^o/ before a final consonant should be inhibited, while a final /a/ should be activated, i.e., {^*^Nom; Gen} → {–^j^o/__C#; +a/__#}. In this case, the inhibitory associations are weaker than the excitatory associations because the deletion of /^j^o/ does not apply every time the –*a* suffix is added.

To underscore the implications of this section for the architecture of grammar, arbitrary alternations can be produced without transformations, exclusively through the use of schematic (form-meaning) associations, as long as we assume 1) that meaning-form associations can be inhibitory (an assumption also needed for truncation and backformation; Kapatsinski, 2021), 2) that activation flow is interactive, and 3) that there is negative feedback from form to message, which allows the speaker to detect a mismatch between the intended message and how the form they are about to produce is likely to be understood. That is, most alternations can in principle be produced by a fully product-oriented system such as one posited by usage-based Construction Grammar (Goldberg, 2002) or Network Theory (Bybee, 1985, 2001), and their mere existence does not threaten the Constructionist Hypothesis or require speakers to learn paradigmatic associations (cf., Pierrehumbert, 2006; Booij, 2010; Becker and Gouskova, 2016; Kapatsinski, 2018a).

Another source of evidence for paradigmatic mappings is that alternations can be produced upon request (e.g., Cappelle, 2006). For example, one could transform *I gave her the book* into *I gave the book to her* when asked for another way to say it. However, stop-signal experiments show that inhibition of a form selected for production can also be produced upon request (Ladefoged et al., 1973). This kind of volitional alternation can be produced by inhibiting the selected production with top-down control, and then allowing activation to spread again from the same message (as proposed by Berg, 1986, for error correction). This process appears sufficient to account for most, if not all, syntactic alternations (Kapatsinski, 2021).

#### The Negative Feedback Cycle in Language Change

We will now briefly consider the implications of the Negative Feedback Cycle for language change. Whereas parallel activation flow from distributed semantic representations provides a mechanism for paradigm leveling (Hoeffner and McClelland, 1993) and grammaticalization (Harmon and Kapatsinski, 2017), the negative feedback cycle provides a mechanism for degrammaticalization and, more generally, deconstructionalization. Consider the classic example of degrammaticalization, *ism* (Ramat, 1992), as in *It is no better than capitalism, socialism or any other “ism” out there*. The message is IDEOLOGY, with a negative connotation. Importantly, the writer intends to transmit the meaning IDEOLOGY without specifying any specific ideology. IDEOLOGY activates words for ideologies, most of which end in *-ism*, such as *capitalism* and *socialism*. However, the stem of each of the activated words activates unintended semantic features, leading to its suppression by the negative feedback cycle. As a result, only *ism* survives.

Another interesting example is presented by deconstructionalization of *pimp my ride* in Dutch. De Pascale et al. (2022) document that Dutch borrowed *pimp my ride* as a fixed expression from an English TV show, but *pimp* was subsequently generalized to other Dutch objects with the meaning FANCIFY. The generalization of *pimp* is particularly puzzling from a usage-based perspective because usage-based models of productivity have proposed that productive patterns (like *pimp*) arise from generalization over experienced instances (Bybee, 1985, 1995; Tomasello, 2003; Goldberg, 2006). However, *pimp* occurred with a single object (*my ride*) when borrowed into Dutch and therefore would not be expected to be productive: there are no objects to generalize over. How then could it gain productivity? The negative feedback cycle provides a possible mechanism: suppose that the speaker wants to express the message FANCIFY+HOUSE. *Pimp my ride* is activated by FANCIFY but *my ride* activates the meaning CAR, which is unintended, and therefore becomes suppressed by negative feedback. This then allows *pimp* to gain autonomy, and to be combined with Dutch objects activated by the message HOUSE.

A related phenomenon explained by the Negative Feedback Cycle is the emergence of libfixes (liberated affixes, which do not correspond to morphemes prior to their liberation, Zwicky, 2010; Norde and Sippach, 2019), as exemplified by the extraction of …[@]*holic* from *alcoholic*. Originally a mere segment sequence within *alcohol-ic*, it can now be productively used to express possession of other addictions (e.g., *workaholic*). Libfixes are formed by backformation, as they do not correspond to morphemes in the original word from which they are extracted. These too likely involve the suppression of a part of a form that is a strong cue to the part of the meaning of the word that the speaker does not intend to express. Of the occurrences of #*alc*… in the Corpus of Contemporary American English, 79% of the tokens are alcohol-related words. No other part of the word is an equally good cue to ALCOHOL (e.g., of the tokens of …*holic*, <10% are alcohol-related). The fact that *alc*- and –[@]*holic* were not traditional morphemes shows that the chunks participating in language production and language change need not align with morpheme boundaries: good cues can both activate a meaning and be suppressed by it when it is unintended. Audring (2021) discusses libfixes as evidence for paradigmatic generalizations (as “emerging solely from speakers recognizing similarities between words,” p. 12). Some libfixes can indeed be accounted for by both generalization over words and the Negative Feedback Cycle. However, cases like –[@]*holic* are only consistent with the Negative Feedback Cycle mechanism because they emerge by extraction from a single word: the open slot in the …ɘ *holic*~ADDICTED.TO… schema could not emerge by generalization because it had a constant filler prior to its emergence.

### The Role of Paradigmatic Associations in Production

The preceding section has argued that much of language production can be accounted for with bidirectional activation flow cycling between message and form. Specifically, it was shown that arbitrary morphophonological alternations can be produced by a feedback cycle in a network of schematic associations. In previous work, such alternations were thought to require paradigmatic mappings turning a source form into a product form, such as k → s (e.g., Pierrehumbert, 2006; Kapatsinski, 2013, 2017a, 2018a; Becker and Gouskova, 2016; Booij and Audring, 2018). This reanalysis therefore raises the question of whether paradigmatic associations are ever necessary.

#### When Paradigmatic Associations Are Necessary

Paradigmatic associations appear to be necessary if an alternation is triggered by a form-level cue that is absent from the output, *as long as that cue is necessary to select between alternative outputs*. An example is presented by suffix choice in Russian Genitives in Table 1. A Russian speaker who accesses the nominative singular form of a Russian noun, or is provided with one in a wug test, can use that form to predict what the genitive form of that same noun is. For example, if the nominative form of the noun ends in *-a*, the speaker should replace the *-a* with *-*ɨ to form the genitive singular. They don't need to know anything else about the noun to do this, and indeed no additional information would be helpful.

**Table 1:** A morphological paradigm that requires paradigmatic associations for productive use.

**Nominative singular**	**Genitive singular**	**Real word example**
xmor.MASC^a^ xmor'.MASC	-a -a	mor “pestilence” xmɨr' “scum”
xmor.FEM xmor'.FEM	0 -i	doris “Doris”^b^ xmar' “gloom”
xmora.MASC xmora.FEM	-i -i	mora “mora” ʒora “George”^c^

**Figure 3:**
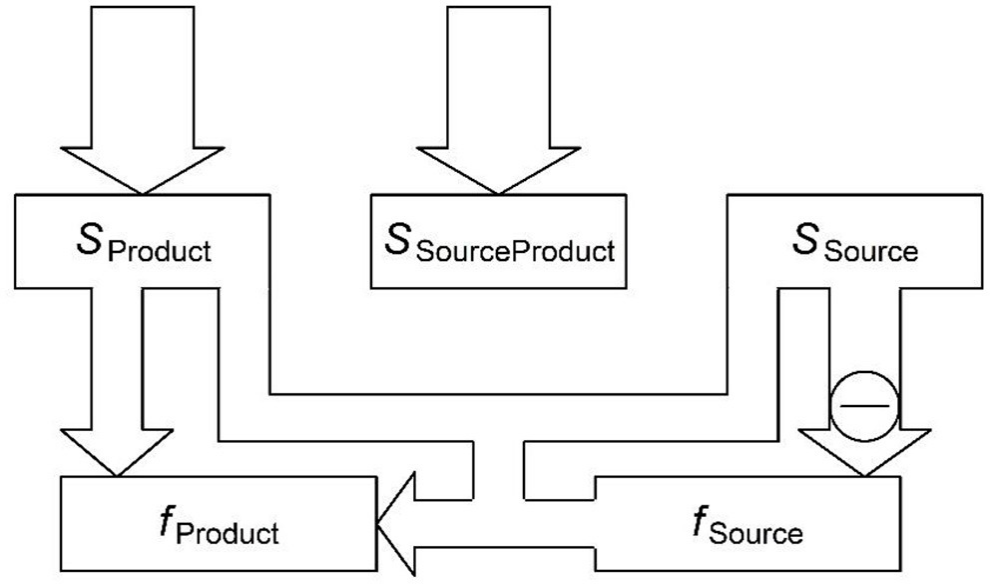
The use of negative feedback and paradigmatic associations to repair a plan that includes a form that does not fully match the intended meaning. The product form is associated with, and receives activation from, the distributed activation pattern specifying a source form, an intended meaning and an unintended meaning. It is also receiving the top-down activation from *S*_Product_ remaining after negative feedback. *S*_SourceProduct_ has been inhibited by the feedback and is no longer sending much activation. *S*_Source_ is sending inhibition down to *f*_Source_.

*The forms in the left column are nonce (“wug”) forms. The middle column shows the suffix that would be attached to them. The right column shows a real-word example of the same pattern. Note that the form of the noun in the Nominative Singular and the meaning are jointly predictive of the correct Genitive form. Hence, the triple-tailed arrow in Figure 3 below*.

a *I assume that MASC and FEM can be considered part of semantics (Boroditsky et al., 2003); see Dilkina et al. (2007) for how these can emerge in a parallel distributed interactive model even for inanimate objects. This is not crucial for the argument*.

b *This type is rare. A borrowed feminine name without a Russian equivalent or an acronym with a feminine head noun are the only common cases*.

c *Most -a-final nouns in Hypochoristics are in this class regardless of gender of the referent, as are many common masculine nouns, like muʒt∫ina ‘man', papa ‘dad', etc*.

This pattern requires paradigmatic associations because it is impossible to determine the form of the genitive without knowing the form of the nominative (i.e., the nominative is a *principal part* of the paradigm). For example, ʒ*ora* is a hypocoristic form of *g*^*j*^*eorg*^*j*^*ij* (“George”), whereas ʒ*or* means “munchies.” The stems of the two forms are homophones. However, because ʒ*ora* ends in the suffix [a] in the Nominative Singular, the Genitive Singular of it is ʒ*ori*. Because ʒ*or* ends in a consonant in the Nominative Singular, the Genitive Singular of it is ʒ*ora*. While all feminines undergo –*a* deletion, any masculine form ending in *-a* (like ʒ*ora*) does too, showing that it is really the phonology of the Nominative that conditions the choice. Because one needs to refer to the *form* of the Nominative to determine the form of the Genitive, a paradigmatic association between the two forms appears necessary, i.e., {…a#~^*^Nom; Gen} → {…i#}. The only hope for a schematic solution is if it can be shown that *semantic* representations discriminate masculine nouns with a nominative *-a* from those that end in a consonant. However, even if they do, it appears implausible that speakers would ignore the far more reliable phonological cue in the nominative source form^8^.

#### Coexistence of Paradigmatic and Schematic Associations

The discussion above suggests that paradigmatic mappings represented by second-order schemas are necessary under rather rare circumstances, which arise only in some languages, suggesting that second-order schemas are typological rarities (Kapatsinski, 2018a). In most cases, productive morphological computation requires nothing more than activation spreading from meaning to form, with the occasional feedback cycle. In contrast, Booij and Audring (2018) suggest that productivity requires a second-order schema. In accordance with a parallel perspective, I suggest that second-order schemas or paradigmatic associations can be used to produce a form even when they are not strictly-speaking necessary, but (conversely) direct form-meaning associations are used for producing forms even when second-order schemas *are* necessary to describe the system. That is, both the direct route, and the indirect, paradigmatic route are active at all times.

Booij and Audring's (2018) proposal that first-order schemas have only a motivational function suggests that production relies exclusively on source-oriented, paradigmatic schemas, whereas judgment can be performed using product-oriented, schematic mappings alone. As a result, the following dissociation can be predicted: a speaker can evaluate a form as being well-motivated, but be unable to produce it, to the extent that its production requires a second-order schema. This dissociation was observed by Smolek and Kapatsinski (2018) in a miniature artificial language learning study, which exposed participants to an articulatorily large typologically rare stem change (p → t∫) unattested in the participants' native language (English). The participants rated plurals with the stem change as being more acceptable than those without (e.g., *but*∫*i* was rated to be a more likely plural form than *bupi*, given the singular *bup*). Yet, they were highly unlikely to produce *but*∫*i* from *bup* in production, usually producing *bupi* instead^9^. Psychological reality of paradigmatic mappings is also suggested by speakers being sensitive to differences in reliability between such mappings, i.e., the probability of the product given the source. Such effects have been observed in child paradigm leveling, where less reliable mappings take longer to learn and are more likely to be leveled (Krajewski et al., 2011; Do, 2013) and in elicited production tests with adults (Pierrehumbert, 2006).

At the same time, a purely paradigmatic approach like Booij and Audring (2018) has trouble accounting for how speakers can derive forms fitting a first-order, product-oriented schema in novel ways that do not fit a second-order schema. For example, the use of product-oriented generalizations in production is suggested by certain patterns of analogical extension, in which a pattern associated with an intended meaning is extended to historically ineligible source forms. In colloquial Russian, the possessive third person pronouns *ix* “their,” *jevo* “his” and *jejo* “her” have served as bases for adjective formation, resulting in the synonyms *ixn*^*j*^*ij* “their,” *jevonnyj* or *jevojnyj* “his,” and *jejnyj* “her,” respectively. There is no source-oriented schema that condones deriving adjectives from possessive pronoun sources. Rather, these derivations suggest an extension of the highly productive product-oriented A~...*nyj*# schema to a new type of source, based on the semantic similarity between adjectives and possessive pronouns, which both serve a restrictive function (i.e., both can be used to answer the question *which N*, as in *which cat? my cat* or *orange cat*). The shared semantics activates the …*nyj*# schema during the production of a possessive adjective. When the …*nyj#* form is produced, and not evaluated negatively, it can take hold, initiating a language change (Harmon and Kapatsinski, 2017).

Productive use of product-oriented schemas is also documented in several nonce probe elicited production studies (wug tests). For example, Wang and Derwing (1994) found that the probability of using a particular stem vowel change to form the past tense in English depended not on the frequency of that change but on the frequency of the resulting vowel in past tense forms. Lobben (1991), in an elicited production experiment with speakers of Hausa, found that product-oriented schemas could be primed, resulting in production of Hausa plurals using stem changes unattested in the lexicon. Kapatsinski (2017a, 2018a: Chapter 7) showed that many English speakers exposed to a miniature language with vowel subtraction in trisyllables (CVCVCV → CVCVC) took these examples to support *addition* of a consonant to disyllables (CVCV → CVCVC); see also Kapatsinski (2012, 2013). The addition of a consonant to CVCV satisfies the schema PL~#CVCVC# at the cost of disobeying the experienced paradigmatic mapping (final vowel deletion, …C_i_V#_SG_ → …C_i_#_PL_). These findings suggest that learners do not rely solely on paradigmatic mappings for productive morphology (contra Booij and Audring, 2018). It is also not clear how a purely paradigmatic mappings can account for libfixation: libfix boundaries do not correspond to morpheme boundaries prior to “liberation,” thus the liberation of *holic* is unexplained by the pre-liberation paradigmatic mapping of …C_i_#~…C_i_ic# in *alcohol*~*alcoholic*.

#### Paradigmatic Associations in Parallel With Negative Feedback

Paradigmatic associations can be incorporated into the proposed architecture alongside schematic associations as shown in Figure 3. For example, consider a Russian speaker who knows a Nominative form of a word, which ends in …a# and now needs to produce the less common Genitive form (*S*_Product_ = GEN). *S*_SourceProduct_ activates the known Nominative form (Figure 1). After feedback spreads up from *f*_Source_ (Figure 2), *f*_Source_ is or contains …*a*#, and *S*_Source_ contains –NOM. Thus, the semantic pattern is telling the network that a Nominative form is activated but a Genitive form is intended, thereby indicating that a change to the activated form is needed. The paradigmatic association provides the mapping to implement: …*a*# → …*i*#. The inhibition of NOM eventually suppresses …*a*#, which is a strong cue to that meaning. However, because this suppression is happening *in parallel with* activation flowing from the source form to the product form, the source form can activate the product form before being inhibited completely.

Paradigmatic cues are activated by the semantics shared between the message and the source form. We denote these semantic cues as *s*_++_. Feedforward activation of a source form (outside of a wug test, where it receives additional perceptual activation) is simply $\underset{s_{++}}{\sum }a_{s_{++}}w_{s_{++}\to f_{Source}}$. This is of course the formula in (1) for feedforward activation. The source form will lose some of this activation via negative feedback even as it is sending activation to the product form (Figure 3). Assuming that the two processes have the same timecourse, half of the total top-down inhibition should be subtracted from the activation available to transmit paradigmatically, which reduces it to $a_{f_{Source}}=\left(\underset{s_{++}}{\sum }a_{s_{++1}}w_{s_{++}\to f_{Source}}\right)\times \left(1-0.5\underset{s_{-}}{\sum }\left(a_{s_{-2}}w_{f_{Source}\to s_{-}}\right)\right)$ where *s*_−_ are unintended semantic features.

Multiple source wordforms are typically activated (to some extent) in a parallel system. This is a crucial property of the model because it accounts for multiple motivation of produced forms: i.e., a form can inherit properties from more than one source form (see Steriade, 1999; Bat-El, 2005; Burzio, 2005; Booij, 2010; Kapatsinski, 2021, for examples). Furthermore, there are multiple chunks activated in each such wordform. As a result, the number of *f*_*Source*_ forms can be quite large, and all of them provide activation to the associated product forms through the paradigmatic associations (*w*_*f*_*Source*_→*f*_), requiring an additional summation over source forms: $a_{Source\to f}=\underset{f_{Source}}{\sum }\left(a_{f_{Source}}w_{f_{Source}\to f}\right).$

## Discussion

The present paper has argued for a parallel, distributed, interactive architecture of language production in which distributed representations of messages are mapped onto chunks of form whose semantics overlap with those of the intended message. In this section, I discuss the relationship of the proposed architecture to previous work, and outline limitations and directions for future work.

### Relations to Other Work

An architecture with these characteristics is widely accepted in psycholinguistics (Dell, 1985, 1986; Martin, 2007; Nozari, 2020), and is also adopted in constructionist linguistic theories, which assign a central role to direct form-meaning mappings. These include Network Theory (Bybee, 1985, 2001), Clamoring for Blends (Kapatsinski, 2013), Usage-based Construction Grammar (Goldberg, 1995, 2006), Construction Morphology (Booij, 2010; Booij and Audring, 2018), and Relational Morphology (Jackendoff and Audring, 2016, 2019, 2020). Aside from the commitment to chunking, these assumptions are also shared with the LDL model (Baayen et al., 2019; Chuang et al., 2021a,b; Heitmeier et al., 2021), and surface-oriented constraint-based approaches like Multiple Correspondence Theory (Burzio, 1998, 2005), Functional Phonology (Boersma, 1998), and Bidirectional Optimality Theory (Boersma, 2011). The proposal that specific repairs are triggered by specific patterns of mismatch between the intended message and the message about to be expressed also resembles ideas from constraints-and-repair-strategies approaches to phonology (Paradis, 1987).

There are also current approaches within morphology that do not share these assumptions. For example, Yang (2016) argues that language production involves serial application of a sequence of rules that follows a serial search through a list of exceptions. Serial approaches are, however, inconsistent with the parallel nature of neural processing and are challenged by many of the phenomena that the parallel approach handle with ease, such as extension of accessible forms to neighboring paradigm cells, multiple correspondence effects, productive product-oriented schemas, libfixation, differences in behavior between forced choice and elicited production tasks, semantic priming effects and their interaction with frequency (see also Kapatsinski, 2018b,c).

The principal contribution of the present paper is to spell out some implications of feedback for the production of complex word forms. I have argued that language production requires feedback to initiate or delay execution, and that one reason to delay execution is if the current production plan is likely to activate meanings that the speaker does not intend to transmit. Mismatch with the message is argued to be both detected and repaired via a *negative feedback cycle*. The negative feedback cycle is also proposed in the LDL model (Baayen et al., 2019; Chuang et al., 2021a,b; Heitmeier et al., 2021), although it works slightly differently in LDL. Specifically, the mechanism proposed here does not require comparison of meanings. Instead, forms inhibit the semantic nodes associated with them, and to the extent that these nodes are not receiving excitation from the message, this automatically results in inhibition spreading down to the associated forms. The best-matching form will generally receive the least amount of inhibition because most of the semantic nodes associated with it are receiving excitation from the message.

The proposed mechanism assumes that meanings are differentiated largely by which semantic nodes are “on.” In a spreading activation framework, activation coming into a node (or a link) is multiplied by its value before it spreads further. Therefore, only the “on” nodes would inhibit associated forms. As a result, there is a distinction between forms that have unintended meanings and forms that are merely more general than the intended message. The negative feedback cycle as envisioned here inhibits the former but not the latter. That is, the negative feedback cycle is intended to inhibit forms whose meanings conflict with the intended message more than it inhibits general placeholder forms like *stuff* . During the feedback cycle, unintended semantic richness hurts; not activating all of the intended meaning does not disadvantage a form during the cycle as much as having unintended meanings does. This echoes Goldberg's (1995) intuition that verbs can be incorporated into novel constructions as long as they have *poorer* argument-structure representations than intended by the speaker. For example, the intransitive *sneeze* is acceptable in *I sneezed her a boatload of viruses*. Even though the semantics of *sneeze* do not include a recipient and a theme, it can be produced with these semantics. On the other hand, *give*, which does have a recipient a theme, cannot be used when these semantic roles are missing from the message, as in ^*^I gave.

I have argued that the negative feedback cycle is central to the production of paradigmatic morphology, which often requires the speaker to make modifications to a frequent base form. For example, *talks* or *talked* are extremely similar in meaning to the more frequent *talk*. If the base form is perceived by the speaker to express the intended message, the speaker has no reason not to produce it, if it happens to be accessed before the context-appropriate inflectional variant. There is extensive evidence that this extension of frequent base forms to other, semantically similar contexts frequently happens in language change, resulting in paradigm leveling (Bybee and Brewer, 1980; Tiersma, 1982; Albright, 2008). To avoid extending a highly accessible form, the speaker needs to detect that it would not be appropriate to the context, and this detection should lead to the form's suppression. The negative feedback cycle accomplishes this task, while parallel processing allows the form to simultaneously activate a context-appropriate alternative, if it is associated with one. The negative feedback cycle makes reference to paradigmatic mappings or rules unnecessary to describe most alternations, except when the speaker needs to refer to a form they do not produce in order to generate the form they do produce.

### Future Directions

The negative feedback cycle makes several interesting predictions. First, it suggests that adjusting forms to avoid overextensions of allomorphs takes time. Some evidence for this prediction has recently been provided by Scheer and Mathy (2021). More generally, the cycle predicts that processing time is needed to avoid ambiguity. There is some evidence consistent with this prediction, in that slower processors (particularly, children) show more overextension. Children with developmental language delay show even slower processing than typically-developing children, and are especially likely to produce overextensions leveling morphological paradigms in favor of frequent forms (Hoeffner and McClelland, 1993; Freudenthal et al., 2021; Harmon et al., 2021). Crucially, overextensions can be reduced by injecting activation into the alternative forms by presenting them to the child in a forced-choice task (Schwab et al., 2018). Because a forced choice presentation feeds activation directly into the feedback cycle, rather than requiring activation to first spread from the message to the alternative forms, it should help the cycle complete more quickly. However, children differ from adults in more than processing speed (e.g., Ramscar et al., 2013). Manipulating the time available before the onset of execution within the same population of participants would be a much stronger test of the hypothesis.

The negative feedback cycle suggests several influences on the likelihood of extending a form to a particular novel semantic context. Because these influences are attributed to the feedback cycle, and not the feedforward activation flow, they are predicted to occur only when the speaker has enough processing time (Dell, 1985). The feedback cycle is most effective at suppressing forms that activate many semantic features not present in the message. That is, unintended semantic richness hurts during the feedback cycle. The negative feedback cycle is relatively ineffective at preventing extension of vague forms. Interestingly, the features that are part of the message but not the meaning of the form are not expected to contribute to the mismatch, even though they are expected to favor the fully-matching competitor (if any) based on feedforward activation flow. In contrast, the feedback cycle guards against extension of forms that have frequent unintended meanings. In other words, it guards against an unintended double entendre: it can inhibit forms that match the intended meaning, but *also* cue a different meaning, especially if they cue it more strongly than the intended meaning.

This predicted avoidance of ambiguity can explain some morphological paradigm gaps. For example, in Russian, the first person singular non-past forms of some verbs appear to be unacceptable because they are shared with more frequent verbs. For example, one of the commonly cited gaps is the first person singular non-past form of the rare verb *d*^*j*^*erz*^*j*^*it*^*j*^ “answer back impolitely,” which would be expected to be *d*^*j*^*er*ʒ*u*. From the negative feedback perspective, it is not an accident that the same form is perfectly acceptable as the first-person singular non-past form of the frequent verb *d*^*j*^*er*ʒ*at*^*j*^ “to hold.” If generated, the form *d*^*j*^*er*ʒ*u* activates HOLD much more than it activates TALK.BACK, because *d*^*j*^*er*ʒ*at*^*j*^ is at least 1,000 times more frequent than *d*^*j*^*erz*^*j*^*it*^*j*^ (Google book ngrams). In contrast, *vo*ʒ*u* is acceptable as a form of both *vod*^*j*^*it*^*j*^ “to lead by hand/leash, or to drive a vehicle” and *voz*^*j*^*it*^*j*^ “to drive a passenger (or cargo),” because the two verbs are about equally frequent, and about 10 times less frequent than *d*^*j*^*er*ʒ*at*^*j*^ (Google book ngrams). Avoidance of homonymy in morphophonological production has also been shown experimentally by Yin and White (2018). However, the role of frequency of the unintended meaning and its interaction with processing time available have not been investigated experimentally.

The negative feedback cycle may be one reason for mismatches between vernacular production and considered judgment. Speakers will often produce forms that they would, upon reflection, judge unacceptable (Labov, 1996). This can happen because the negative feedback cycle will not always complete before a form is sent to execution. The feedback cycle suggests, as Labov has also argued, that stigmatized productions are likely to slip through when the speaker's attention is drawn away from stylistic connotations, i.e., *s*_Source_. It also predicts that such variants are likely to slip through if the speaker is under time pressure.

In morphology, paradigm gaps present a particularly interesting example of stigmatized variants slipping through. A paradigm gap refers to the situation in which no form of a particular word is perceived as an acceptable filler for a particular paradigm cell. For example, as mentioned earlier. *d*^*j*^*erz*^*j*^*it*^*j*^ “answer back impolitely” has no 1^st^ person singular non-past. There is much debate about the reasons for paradigm gaps, and none of the explanations (including avoidance of ambiguity) explain all gapped forms. Furthermore, the ostensibly unacceptable forms are indeed produced in casual writing (Kulinich, 2020). Therefore, speakers need to remember to avoid producing certain forms in formal contexts (Daland et al., 2007). By distinguishing between an initial stage of processing that generates multiple competing alternatives, and a subsequent stage in which these forms are suppressed by negative feedback, the negative feedback cycle explains how paradigm gaps can look like variation in everyday, casual language production, while generating distinctly different reactions in considered judgment, and being avoided in monitored speech and writing. The negative nature of the proposed feedback mechanism makes it particularly useful for explaining gaps.

### Limitations

Some aspects of the architecture remain underspecified. In particular, I did not discuss how order is represented within the production plan, and how the chunks selected for production are fitted together into a word form. When do co-occurring forms fuse into a chunk (or, conversely, when do chunks split into associated forms)? More generally, how are the chunks and association weights learned? How do the proposed mechanisms map onto the brain? How is selection accomplished, e.g., *via* deterministically selecting the most activated form, or matching choice probabilities to activations? Most importantly, the formulas above are only approximations to reality because they make simplifying assumptions to which the theory is not committed. In particular, they assume a sequencing of the steps shown in Figures 1–3 even though activation cascades continuously through an interactive network. The dynamics of activation spread in an interactive activation model can be quite complex and the resulting distribution of activation over nodes in general cannot be determined analytically. Although there are implemented models that address many of the issues above (see esp., McCauley and Christiansen, 2019; Heitmeier et al., 2021), they have not combined chunking with interactive activation flow. Simulation of the architecture therefore constitutes the major task for future work.

## Data Availability Statement

The original contributions presented in the study are included in the article/supplementary material, further inquiries can be directed to the corresponding author/s.

## Author Contributions

VK was responsible for all aspects of this manuscript.

## Conflict of Interest

The author declares that the research was conducted in the absence of any commercial or financial relationships that could be construed as a potential conflict of interest.

## Publisher's Note

All claims expressed in this article are solely those of the authors and do not necessarily represent those of their affiliated organizations, or those of the publisher, the editors and the reviewers. Any product that may be evaluated in this article, or claim that may be made by its manufacturer, is not guaranteed or endorsed by the publisher.

## Footnotes

^1^Note that I do not wish to commit to how coarse-grained semantic representations are. Thus, the activated nodes can be thought of as either semantic (micro)features referring to individual neurons or concept ‘pointer' nodes that group together frequently coactivated nodes from across the cortex.

^2^The Constructionist Hypothesis is perceived to be in danger. For example, the latest International Conference on Construction Grammar (2021) held two parallel workshops on the topic of how paradigmatic mappings could be incorporated into the constructionist framework.

^3^In some situations, such as elicited production tests (Berko, 1958), where the source form is provided to the speaker, the source form *f*_Source_ also receives input activation (from perception) and would have a sourceless arrow pointing to it, predicting that leveling of stem changes is particularly likely in such paradigms. Note that the product and source forms can be both lexical and sublexical, and that there is often more than one source form.

^4^Following Jamieson et al. (2012), I assume that the spreading activation/inhibition does not change the ‘content' of the semantic nodes (0 or 1). It merely changes the activation level of that content. Because of this, feedback does not change word meanings.

^5^A counterintuitive prediction is that product forms with unique semantics not shared with source forms are disfavored during negative feedback, compared to product forms that have more semantic overlap with source forms. However, this appears unproblematic because forms with unique semantics face little semantic competition in the initial feedforward stage in Figure 1 and so are unlikely to need feedback to be selected.

^6^This assumes that $a_{s_{1}}>\underset{f}{\sum }\left(w_{f\to s}a_{f_{1}}\right)$. This may not be the case if there are many alternative activated forms that share semantics with the message. This is one way that the system can produce a paradigm gap / lead the speaker to freeze in uncertainty.

^7^The *w*_*f*→*s*_ rather than *w*_*s*→*f*_ in this formula assumes that the negative feedback cycle is most effective at suppressing forms that strongly cue unintended meanings rather than the forms most strongly cued by them (as in LDL; Chuang et al., 2021b). Implementing this mechanism with activation flow relies on the possibility of bidirectional schematic associations learned by predicting meaning from forms (Kapatsinski, 2021).

^8^As noted above, LDL rejects the use of paradigmatic form cues, and Chuang et al. (2020) argue that LDL is able to account for the complex morphology of Estonian without reference to principal part forms. However, the model's performance on novel words is far from the ceiling (45%), which suggests that there is room for the addition of paradigmatic form cues to improve the model's prediction accuracy.

^9^No dissociation was observed for the changes t → t∫ and k → t∫, which were judged to be acceptable about as often as they were produced. This suggests that second-order schemas may be necessary specifically to enact large changes. However, production of these changes could rely on paradigmatic mappings learned from exposure to English (e.g., *crea*[t]*e*~*crea*[t∫]*ure, fun*[dʒi]~*fun*[g]*al*).

## References

[B1] Abdel Rahman R. Aristei S. (2010). Now you see it… and now again: Semantic interference reflects lexical competition in speech production with and without articulation. Psychon. Bull. Rev. 17, 657–661. 10.3758/PBR.17.5.657 21037163

[B2] Albright A. (2008). “Explaining universal tendencies and language particulars in analogical change,” in Linguistic Universals and Language Change, ed J. Good (Oxford: Oxford University Press). 10.1093/acprof:oso/9780199298495.003.0007

[B3] Albright A. Hayes B. (2003). Rules vs. analogy in English past tenses: A computational/experimental study. Cognition 90, 119–161. 10.1016/S0010-0277(03)00146-X 14599751

[B4] Ambridge B. Lieven E. V. (2011). Child Language Acquisition: Contrasting Theoretical Approaches. Cambridge: Cambridge University Press. 10.1017/CBO9780511975073

[B5] Aronoff M. (1976). Word Formation in Generative Grammar. Cambridge, MA: MIT Press.

[B6] Audring J. (2021). Advances in morphological theory: construction morphology and relational morphology. Ann. Rev. Linguistics 8:115118. 10.1146/annurev-linguistics-031120-115118

[B7] Baayen R. H. Chuang Y. Y. Shafaei-Bajestan E. Blevins J. P. (2019). The discriminative lexicon: A unified computational model for the lexicon and lexical processing in comprehension and production grounded not in (de)composition but in linear discriminative learning. Complexity 2019:5891. 10.1155/2019/4895891

[B8] Baayen R. H. Hendrix P. (2016). “Two-layer networks, non-linear separation, and human learning,” in From Semantics to Dialectometry, eds M. Wieling, G. Bouma, and G. van Noord (Groningen: University of Groningen).

[B9] Barros-Loscertales A. González J. Pulvermüller F. Ventura-Campos N. Bustamante J. C. Costumero V.. (2012). Reading *salt* activates gustatory brain regions: fMRI evidence for semantic grounding in a novel sensory modality. Cerebral Cortex 22, 2554–2563. 10.1093/cercor/bhr324 22123940 PMC4705335

[B10] Bat-El O. (2005). “Competing principles of paradigm uniformity: Evidence from the Hebrew imperative paradigm,” in Paradigms in Phonological Theory, eds L. J. Downing, T. A. Hall, and R. Raffelsiefen (Oxford: Oxford University Press). 10.1093/acprof:oso/9780199267712.003.0003

[B11] Becker M. Gouskova M. (2016). Source-oriented generalizations as grammar inference in Russian vowel deletion. Linguistic Inquiry 47, 391–425. 10.1162/LING_a_00217

[B12] Beniaguev D. Segev I. London M. (2021). Single cortical neurons as deep artificial neural networks. Neuron 109, 2727–2739. 10.1016/j.neuron.2021.07.002 34380016

[B13] Benua L. (1997). Transderivational Identity. (Doctoral dissertation, University of Massachusetts, Amherst).

[B14] Berg T. (1986). The problems of language control: Editing, monitoring, and feedback. Psychol. Res. 48, 133–144. 10.1007/BF00309161

[B15] Berko J. (1958). The child's learning of English morphology. Word 14, 150–177. 10.1080/00437956.1958.11659661

[B16] Bock K. Griffin Z. M. (2000). The persistence of structural priming: Transient activation or implicit learning? J. Experi. Psychol. Gene. 129, 177–192. 10.1037/0096-3445.129.2.177 10868333

[B17] Boersma P. (1998). Functional Phonology. (Doctoral dissertation, Netherlands Graduate School of Linguistics).

[B18] Boersma P. (2011). “A programme for bidirectional phonology and phonetics and their acquisition and evolution,” in Bidirectional Optimality Theory, eds A. Benz and J. Mattausch (Amsterdam: John Benjamins). 10.1075/la.180.02boe

[B19] Booij G. (1997). Autonomous Morphology and Paradigmatic Relations. Yearbook of Morphology. Springer. 10.1007/978-94-017-3718-0_4

[B20] Booij G. (2010). Construction Morphology. Oxford: Oxford University Press. 10.1093/oxfordhb/9780199695720.013.0010

[B21] Booij G. Audring J. (2018). “Partial motivation, multiple motivation: The role of output schemas in morphology,” in The Construction of Words: Advances in Construction Morphology, ed G. Booij (Cham: Springer). 10.1007/978-3-319-74394-3_3

[B22] Boroditsky L. Schmidt L. Phillips W. (2003). “Sex, Syntax, and Semantics,” in Language in Mind: Advances in the Study of Language and Cognition, eds D. Gentner and S. Goldin-Meadow (Cambridge, MA: MIT Press).

[B23] Botvinick M. M. Braver T. S. Barch D. M. Carter C. S. Cohen J. D. (2001). Conflict monitoring and cognitive control. Psychol. Rev. 108, 624–652. 10.1037/0033-295X.108.3.624 11488380

[B24] Braine M. D. Brody R. E. Brooks P. J. Sudhalter V. Ross J. A. Catalano L.. (1990). Exploring language acquisition in children with a miniature artificial language: Effects of item and pattern frequency, arbitrary subclasses, and correction. J. Mem. Lang. 29, 591–610. 10.1016/0749-596X(90)90054-4

[B25] Breiss C. (2021). Lexical Conservatism in Phonology: Theory, Experiments, and Computational Modeling (Doctoral dissertation, UCLA).

[B26] Burzio L. (1998). Multiple correspondence. Lingua 103, 79–109. 10.1016/S0024-3841(97)00025-9

[B27] Burzio L. (2005). “Sources of paradigm uniformity,” in Paradigms in Phonological Theory, eds L. J. Downing, T. A. Hall, and R. Raffelsiefen (Oxford: Oxford University Press). 10.1093/acprof:oso/9780199267712.003.0004

[B28] Bybee J. (1985). Morphology: A Study in the Relation Between Meaning and Form. Amsterdam: John Benjamins. 10.1075/tsl.9

[B29] Bybee J. (1995). Regular morphology and the lexicon. Lang. Cogn. Processes. 10, 425–455.

[B30] Bybee J. (2001). Phonology and Language Use. Cambridge: Cambridge University Press. 10.1017/CBO9780511612886

[B31] Bybee J. (2003). “Cognitive processes in grammaticalization,” in The New Psychology of Language, ed M. Tomasello (Psychology Press). 10.4324/9781410606921-8

[B32] Bybee J. (2006). From usage to grammar: The mind's response to repetition. Language 82, 711–733. 10.1353/lan.2006.0186

[B33] Bybee J. (2015). Language Change. Cambridge: Cambridge University Press. 10.1017/CBO9781139096768

[B34] Bybee J. L. Brewer M. A. (1980). Explanation in morphophonemics: Changes in Provençal and Spanish preterite forms. Lingua 52, 201–242. 10.1016/0024-3841(80)90035-2

[B35] Bybee J. L. McClelland J. L. (2005). Alternatives to the combinatorial paradigm of linguistic theory based on domain-general principles of human cognition. Linguistic Rev. 22, 381–410. 10.1515/tlir.2005.22.2-4.381

[B36] Bybee J. L. Perkins R. D. Pagliuca W. (1994). The Evolution of Grammar: Tense, Aspect, and Modality in the Languages of the World. Chicago, IL: University of Chicago Press.

[B37] Caballero G. Kapatsinski V. (2022). “How agglutinative? Searching for cues to meaning in Choguita Rarámuri (Tarahumara) using discriminative learning,” in Morphological Diversity and Linguistic Cognition, eds A. Sims, A. Ussishkin, J. Parker, and S. Wray (Cambridge: Cambridge University Press).

[B38] Cappelle B. (2006). Particle placement and the case for 'allostructions. Constructions 1, 1–28.

[B39] Chuang Y. Y. Bell M. J. Banke I. Baayen R. H. (2021a). Bilingual and multilingual mental lexicon: A modeling study with Linear Discriminative Learning. Lang. Learn. 71, 219–292. 10.1111/lang.12435

[B40] Chuang Y. Y. Lõo K. Blevins J. P. Baayen R. H. (2020). “Estonian case inflection made simple. A case study in Word and Paradigm morphology with Linear Discriminative Learning,” in Complex Words: Advances in Morphology, eds L. Körtvélyessy and P. Štekauer (Cambridge: Cambridge University Press). 10.1017/9781108780643.008

[B41] Chuang Y. Y. Vollmer M. L. Shafaei-Bajestan E. Gahl S. Hendrix P. Baayen R. H. (2021b). The processing of pseudoword form and meaning in production and comprehension: A computational modeling approach using linear discriminative learning. Behav. Res. Methods 53, 945–976. 10.3758/s13428-020-01356-w 32377973 PMC8219637

[B42] Daland R. Sims A. D. Pierrehumbert J. (2007). Much ado about nothing: A social network model of Russian paradigmatic gaps. Proc. Ann. Meeting Assoc. Comput. Linguistics 45, 936–943.

[B43] De Pascale S. Pijpops D. Van de Velde F. Zenner E. (2022). Reassembling the pimped ride: A quantitative look at the integration of a borrowed expression. Front. Commun. 10.3389/fcomm.2022.777312

[B44] Dell G. S. (1985). Positive feedback in hierarchical connectionist models: Applications to language production. Cogn. Sci. 9, 3–23. 10.1207/s15516709cog0901_2

[B45] Dell G. S. (1986). A spreading-activation theory of retrieval in sentence production. Psychol. Rev. 93, 283–321. 10.1037/0033-295X.93.3.283 3749399

[B46] Diessel H. (2019). The Grammar Network. How Linguistic Structure Is Shaped by Language Use. Cambridge: Cambridge University Press. 10.1017/9781108671040

[B47] Diessel H. (2020). A dynamic network approach to the study of syntax. Front. Psychol. 11, e604853. 10.3389/fpsyg.2020.604853 33329277 PMC7732467

[B48] Dilkina K. McClelland J. L. Boroditsky L. (2007). How language affects thought in a connectionist model. Proc. Ann. Meet. Cogn. Sci. Soc. 29, 215–220.

[B49] Do Y. A. (2013). Biased Learning of Phonological Alternations. (Doctoral dissertation, MIT).

[B50] Downing L. J. Hall T. A. Raffelsiefen R. (2005). “Introduction,” in Paradigms in Phonological Theory, eds L. J. Downing, T. A. Hall, and R. Raffelsiefen (Oxford: Oxford University Press). 10.1093/acprof:oso/9780199267712.003.0001

[B51] Ervin S. M. (1961). Changes with age in the verbal determinants of word-association. Am. J. Psychol. 74, 361–372. 10.2307/1419742 13697296

[B52] Falandays J. B. Nguyen B. Spivey M. J. (2021). Is prediction nothing more than multi-scale pattern completion of the future? Brain Res. 2021:147578. 10.1016/j.brainres.2021.147578 34284021

[B53] Ferreira V. S. Griffin Z. M. (2003). Phonological influences on lexical (mis) selection. Psychol. Sci. 14, 86–90. 10.1111/1467-9280.01424 12564760

[B54] Ford A. Singh R. Martohardjono G. (1997). Pace Panini: Towards a Word-Based Theory of Morphology. New York, NY: Lang.

[B55] French R. M. Addyman C. Mareschal D. (2011). TRACX: A recognition-based connectionist framework for sequence segmentation and chunk extraction. Psychol. Rev. 118, 614–636. 10.1037/a0025255 22003842

[B56] Freudenthal D. Ramscar M. Leonard L. B. Pine J. M. (2021). Simulating the acquisition of verb inflection in Typically Developing children and children with Developmental Language Disorder in English and Spanish. Cogn. Sci. 45:e12945. 10.1111/cogs.12945 33682196

[B57] Goldberg A. E. (1995). Constructions: A Construction Grammar Approach to Argument Structure. Chicago, IL: University of Chicago Press.

[B58] Goldberg A. E. (2002). Surface generalizations: An alternative to alternations. Cogn. Linguist. 13, 327–356. 10.1515/cogl.2002.022

[B59] Goldberg A. E. (2006). Constructions at Work: The Nature of Generalization in Language. Oxford: Oxford University Press.

[B60] Hale M. Kissock M. Reiss C. (1998). Output-output correspondence in optimality theory. Proc. West Coast Conf. Formal Linguistics 16, 223–236.

[B61] Harmon Z. Barak L. Shafto P. Edwards J. Feldman N. H. (2021). Making heads or tails of it: a competition–compensation account of morphological deficits in language impairment. Proc. Ann. Meet. Cogn. Sci. Soc. 43, 1872–1878.

[B62] Harmon Z. Kapatsinski V. (2015). Studying the dynamics of lexical access using disfluencies. Proc. Disfluen. Spontaneous Speech 2015, 41–44.

[B63] Harmon Z. Kapatsinski V. (2017). Putting old tools to novel uses: The role of form accessibility in semantic extension. Cogn. Psychol. 98, 22–44. 10.1016/j.cogpsych.2017.08.002 28830015

[B64] Harmon Z. Kapatsinski V. (2021). A theory of repetition and retrieval in language production. Psychol. Rev. 128, 1112–1144. 10.1037/rev0000305 34242049

[B65] Hauk O. Davis M. H. Kherif F. Pulvermüller F. (2008). Imagery or meaning? Evidence for a semantic origin of category-specific brain activity in metabolic imaging. Eur. J. Neurosci. 27, 1856–1866. 10.1111/j.1460-9568.2008.06143.x 18380676 PMC2327213

[B66] Heine B. Kuteva T. (2002). World Lexicon of Grammaticalization. Cambridge, UK: Cambridge University Press. 10.1017/CBO9780511613463

[B67] Heitmeier M. Chuang Y.-Y. Baayen R. H. (2021). Modeling morphology with Linear Discriminative Learning: Considerations and design choices. Arxiv preprint, arXiv:2106.07936. 10.3389/fpsyg.2021.720713 34867600 PMC8634146

[B68] Herzog S. Tetzlaff C. Wörgötter F. (2020). Evolving artificial neural networks with feedback. Neural Networks 123, 153–162. 10.1016/j.neunet.2019.12.004 31874331

[B69] Hinton G. E. (1981). “Implementing semantic networks in parallel hardware,” in Parallel Models of Associative Memory, eds G. E. Hinton and J. A. Anderson (Hillsdale, NJ: Erlbaum).

[B70] Hoeffner J. H. McClelland J. L. (1993). Can a perceptual processing deficit explain the impairment of inflectional morphology in development dysphasia? A computational investigation. Proc. Ann. Child Lang. Res. Forum 25, 38–49.

[B71] Hoenig K. Sim E. J. Bochev V. Herrnberger B. Kiefer M. (2008). Conceptual flexibility in the human brain: Dynamic recruitment of semantic maps from visual, motor, and motion-related areas. J. Cogn. Neurosci. 20, 1799–1814. 10.1162/jocn.2008.20123 18370598

[B72] Holler J. Alday P. M. Decuyper C. Geiger M. Kendrick K. H. Meyer A. S. (2021). Competition reduces response times in multiparty conversation. Front. Psychol. 12, e693124. 10.3389/fpsyg.2021.693124 34603124 PMC8481383

[B73] Jackendoff R. Audring J. (2016). Morphological schemas: Theoretical and psycholinguistic issues. Ment. Lex. 11, 467–493. 10.1075/ml.11.3.06jac

[B74] Jackendoff R. Audring J. (2019). The Texture of the Lexicon: Relational Morphology and the Parallel Architecture. Oxford: Oxford University Press. 10.1093/oso/9780198827900.001.0001

[B75] Jackendoff R. Audring J. (2020). Relational morphology: a cousin of construction grammar. Front. Psychol. 11, 2241. 10.3389/fpsyg.2020.02241 33071852 PMC7538549

[B76] Jamieson R. K. Crump M. J. Hannah S. D. (2012). An instance theory of associative learning. Learn. Behav. 40, 61–82. 10.3758/s13420-011-0046-2 21913057

[B77] Janda R. D. (2000). Beyond “pathways” and “unidirectionality”: On the discontinuity of language transmission and the counterability of grammaticalization. Lang. Sci. 23, 265–340. 10.1016/S0388-0001(00)00023-1

[B78] Kapatsinski V. (2012). “What statistics do learners track? Rules, constraints and schemas in (artificial) grammar learning,” in Frequency Effects in Language Learning and Processing, eds S. Th. Gries and D. Divjak (Berlin: Mouton de Gruyter). 10.1515/9783110274059.53

[B79] Kapatsinski V. (2013). Conspiring to mean: Experimental and computational evidence for a usage-based harmonic approach to morphophonology. Language 89, 110–148. 10.1353/lan.2013.0003

[B80] Kapatsinski V. (2017a). Learning a subtractive morphological system: Statistics and representations. Proc. Boston University Conf. Lang. Dev. 41, 357–372.

[B81] Kapatsinski V. (2017b). “Copying, the source of creativity,” in Each Venture a New Beginning: Studies in Honor of Laura A. Janda, eds A. Makarova, S. M. Dickey, and D. Divjak (Bloomington, IN: Slavica).

[B82] Kapatsinski V. (2018a). Changing Minds Changing Tools: From Learning Theory to Language Acquisition to Language Change. Cambridge, MA: MIT Press. 10.7551/mitpress/11400.001.0001

[B83] Kapatsinski V. (2018b). On the intolerance of the Tolerance Principle. Linguistic Approaches to Bilingualism 8, 738–742. 10.1075/lab.18052.kap

[B84] Kapatsinski V. (2018c). “Words versus rules (Storage versus online production/processing) in morphology,” in Oxford Research Encyclopedia of Linguistics, ed M. Aronoff (Oxford: Oxford University Press). 10.1093/acrefore/9780199384655.013.598

[B85] Kapatsinski V. (2021). What are constructions, and what else is out there? An associationist perspective. Front. Commun. 5, 134. 10.3389/fcomm.2020.575242

[B86] Kapatsinski V. Harmon Z. (2017). Hebbian account of entrenchment and (over)-extension in language learning. Proc. Ann. Meet. Cogn. Sci. Soc. 39, 2366–2371.

[B87] Koranda M. Zettersten M. MacDonald M. C. (2018). Word frequency can affect what you choose to say. Proc. Ann. Meet. Cogn. Sci. Soc. 40, 629–634.

[B88] Krajewski G. Theakston A. L. Lieven E. V. Tomasello M. (2011). How Polish children switch from one case to another when using novel nouns: challenges for models of inflectional morphology. Lang. Cogn. Process. 26, 830–861. 10.1080/01690965.2010.506062

[B89] Kulinich E. (2020). Does the Tolerance Principle Explain the Problem of Russian Paradigm Gaps? Poster Presented at the Annual Meeting of the Canadian Linguistics Association. Available online at: https://cla-acl.ca/pdfs/affiches-2020/2020-CLA-poster-Kulinich.pdf

[B90] Labov W. (1996). When intuitions fail. Proc. Chicago Linguistic Soc. 32, 77–106.

[B91] Ladefoged P. Silverstein R. Papcun G. (1973). Interruptibility of speech. J. Acoustical Soc. Am. 54, 1105–1108. 10.1121/1.1914323 4757456

[B92] Langacker R. W. (1987). Foundations of cognitive grammar. Vol.1: Theoretical prerequisites. Stanford, CA: Stanford University Press.

[B93] Lobben M. (1991). Pluralization of Hausa Nouns, Viewed From Psycholinguistic Experiments and Child Language Data. (MA thesis, University of Oslo).

[B94] Maess B. Friederici A. D. Damian M. Meyer A. S. Levelt W. J. (2002). Semantic category interference in overt picture naming: Sharpening current density localization by PCA. J. Cogn. Neurosci. 14, 455–462. 10.1162/089892902317361967 11970804

[B95] Magnuson J. Grubb S. Crinnion A. M. Luthra S. Gaston P. (2021). Contra assertions, feedback improves word recognition. PsyArxiv preprint. 10.31234/osf.io/aq2cx PMC11238470 37944313

[B96] Marsolek C. J. Schnyer D. M. Deason R. G. Ritchey M. Verfaellie M. (2006). Visual antipriming: Evidence for ongoing adjustments of superimposed visual object representations. Cogn. Affect. Behav. Neurosci. 6, 163–174. 10.3758/CABN.6.3.163 17243353

[B97] Martin A. T. (2007). The Evolving Lexicon. (Doctoral dissertation, UCLA).

[B98] McCauley S. M. Christiansen M. H. (2019). Language learning as language use: A cross-linguistic model of child language development. Psychol. Rev. 126, 1–51. 10.1037/rev0000126 30604987

[B99] McClelland J. L. (1981). Retrieving general and specific information from stored knowledge of specifics. Proc. Ann. Meet. Cogn. Sci. Soc. 3, 170–172.

[B100] McClelland J. L. Elman J. L. (1986). The TRACE model of speech perception. Cogn. Psychol. 18, 1–86. 10.1016/0010-0285(86)90015-0 3753912

[B101] McRae K. De Sa V. R. Seidenberg M. S. (1997). On the nature and scope of featural representations of word meaning. J. Experi. Psychol. 126, 99–130. 10.1037/0096-3445.126.2.99 9163932

[B102] Meyer A. S. (1996). Lexical access in phrase and sentence production: Results from picture–word interference experiments. J. Mem. Lang. 35, 477–496. 10.1006/jmla.1996.0026

[B103] Motley M. T. Camden C. T. Baars B. J. (1982). Covert formulation and editing of anomalies in speech production: Evidence from experimentally elicited slips of the tongue. J. Verbal Learn. Verbal Behav. 21, 578–594. 10.1016/S0022-5371(82)90791-5

[B104] Nesset T. (2008). Abstract Phonology in a Concrete Model. Berlin: De Gruyter Mouton. 10.1515/9783110208368

[B105] Newmeyer F. J. (1998). Language Form and Language Function. Cambridge, MA: MIT Press.

[B106] Norde M. Sippach S. (2019). Nerdalicious scientainment: A network analysis of English libfixes. Word Struct. 12, 353–384. 10.3366/word.2019.0153

[B107] Nozari N. (2020). A comprehension-or a production-based monitor? Response to Roelofs 2020. J. Cognit. 3:19. 10.5334/joc.102 32944682 PMC7473204

[B108] Nozari N. Dell G. S. Schwartz M. F. (2011). Is comprehension the basis for error detection? A conflict-based theory of error detection in speech production. Cogn. Psychol. 63, 1–33. 10.1016/j.cogpsych.2011.05.001 21652015 PMC3135428

[B109] O'Donnell T. J. (2015). Productivity and Reuse in Language: A Theory of Linguistic Computation and Storage. Cambridge, MA: MIT Press. 10.7551/mitpress/9780262028844.001.0001

[B110] Osthoff H. Brugman K. (1878). Morphologische Untersuchungen auf dem Gebiete der indogermanischen Sprachen. Part I. Leipzig: Hirzel.

[B111] Paradis C. (1987). On constraints and repair strategies. Linguistic Rev. 6, 71–97. 10.1515/tlir.1987.6.1.71

[B112] Perruchet P. Vintner A. (1998). PARSER: A model for word segmentation. J. Mem. Lang. 39, 246–263. 10.1006/jmla.1998.2576

[B113] Pierrehumbert J. B. (2006). “The statistical basis of an unnatural alternation,” in Laboratory Phonology, eds L. Goldstein, D. H. Whalen, and C. T. Best (Berlin: De Gruyter Mouton).

[B114] Pinet S. Nozari N. (2018). “Twisting fingers”: The case for interactivity in typed language production. Psychon. Bull. Rev. 25, 1449–1457. 10.3758/s13423-018-1452-7 29687398

[B115] Rabovsky M. Schad D. J. Abdel Rahman R. (2016). Language production is facilitated by semantic richness but inhibited by semantic density: Evidence from picture naming. Cognition 146, 240–244. 10.1016/j.cognition.2015.09.016 26468758

[B116] Ramat P. (1992). Thoughts on degrammaticalization. Linguistics 30, 549–560. 10.1515/ling.1992.30.3.549

[B117] Ramscar M. Dye M. McCauley S. M. (2013). Error and expectation in language learning: The curious absence of “mouses” in adult speech. Language 89, 760–793. 10.1353/lan.2013.0068

[B118] Rogers T. T. McClelland J. L. (2004). Semantic cognition: A parallel distributed processing approach. Cambridge, MA: MIT press. 10.1038/nrn1076 12671647

[B119] Rose S. B. Aristei S. Melinger A. Abdel Rahman R. (2019). The closer they are, the more they interfere: Semantic similarity of word distractors increases competition in language production. J. Experi. Psychol. 45, 753–763. 10.1037/xlm0000592 29975074

[B120] Scheer T. Mathy F. (2021). Neglected factors bearing on reaction time in language production. Cogn. Sci. 45:13050. 10.1111/cogs.13050 34643964

[B121] Schmid H.-J. (2020). The Dynamics of the Linguistic System: Usage, Conventionalization, and Entrenchment. Oxford: Oxford University Press. 10.1093/oso/9780198814771.001.0001

[B122] Schnadt M. J. (2009). Lexical Influences on Disfluency Production. (Doctoral dissertation, University of Edinburgh).

[B123] Schwab J. F. Lew-Williams C. Goldberg A. E. (2018). When regularization gets it wrong: Children over-simplify language input only in production. J. Child Lang. 45, 1054–1072. 10.1017/S0305000918000041 29463337 PMC6076332

[B124] Servan-Schreiber E. Anderson J. R. (1990). Learning artificial grammars with competitive chunking. J. Experi. Psychol. 16, 592–608. 10.1037/0278-7393.16.4.59224905545

[B125] Smolek A. Kapatsinski V. (2018). What happens to large changes? Saltation produces well-liked outputs that are hard to generate. Lab. Phonol. 9:93. 10.5334/labphon.93

[B126] Solan Z. Horn D. Ruppin E. Edelman S. (2005). Unsupervised learning of natural languages. Proc. Nat. Acad. Sci. U.S.A. 102, 11629–11634. 10.1073/pnas.0409746102 16087885 PMC1187953

[B127] Steriade D. (1999). Lexical conservatism in French adjectival liaison. Proc. Linguistic Colloquium Romance Lang. 25, 243–270. 10.1075/cilt.185.18ste

[B128] Steriade D. (2000). “Paradigm uniformity and the phonetics-phonology boundary,” in Papers in Laboratory Phonology V: Acquisition and the Lexicon, eds M. B. Broe and J. B. Pierrehumbert (Cambridge: Cambridge University Press).

[B129] Tiersma P. M. (1982). Local and general markedness. Language 58, 832–849. 10.2307/413959

[B130] Tomasello M. (2003). Constructing a Language: A Usage-Based Theory of Language Acquisition. Cambridge, MA: Harvard University Press.

[B131] Tyler L. K. Moss H. E. (2001). Towards a distributed account of conceptual knowledge. Trends Cogn. Sci. 5, 244–252. 10.1016/S1364-6613(00)01651-X 11390295

[B132] Wang H. S. Derwing B. L. (1994). “Some vowel schemas in three English morphological classes: Experimental evidence,” in In Honor of Professor William S.-Y. Wang: Interdisciplinary studies on Language and Language Change, eds M. Y. Chen and O. C. L. Tseng (Taipei: Pyramid Press).

[B133] Yang C. (2016). The Price of Linguistic Productivity: How Children Learn to Break the Rules of Language. Cambridge, MA: MIT Press. 10.7551/mitpress/9780262035323.001.0001

[B134] Yin S. H. White J. (2018). Neutralization and homophony avoidance in phonological learning. Cognition 179, 89–101. 10.1016/j.cognition.2018.05.023 29933120

[B135] Zwicky A. M. (2010). Libfixes. Blog post. Available online at: https://arnoldzwicky.~org/2010/01/23/libfixes/

